# A Re-Audit of Tier 4 Child and Adolescent Admissions Through the Alliance Crisis and Home Treatment Team, West London Mental Health Trust Over a One-Year Period

**DOI:** 10.1192/bjo.2026.11822

**Published:** 2026-06-30

**Authors:** Jade Parkinson, Mariam Hannalla, Umaira Arif, Tania Saour

**Affiliations:** West London NHS Foundation Trust, London, United Kingdom

## Abstract

**Aims:**

To examine trends and identify potential contributing factors among young people who were supported by the Alliance Team prior to admission to a Tier 4 bed over a one-year period.

To examine the impact of Alliance Team involvement in implementing national guidance regarding avoiding unncessary admissions and improving the experience of those for whom admission was unavoidable.

**Methods:**

A retrospective review of the Alliance Team caseload between April 2024 and April 2025 was undertaken. Electronic patient records were reviewed using the West London RiO system. Data collected included age, gender, referral reason, legal status at the time of admission, reason for admission, location and duration of admission, prior community team involvement, Alliance Team involvement in gatekeeping decisions, and recorded diagnoses of Attention Deficit Hyperactivity Disorder (ADHD) or Autism Spectrum Disorder (ASD). Data were collated and descriptively analysed using Microsoft Excel.

**Results:**

During the study period, the Alliance Team received 465 referrals, with 20 young people admitted to Tier 4 services (4.3%). Most admitted patients were female (70%) and aged 17 years (65%). The majority were admitted under the Mental Health Act, with only one informal admission. 65% were admitted to local beds. The most common reason for admission was risk to self that could not be managed in the community, followed by psychosis. Admission duration varied, with 35% staying less than 30 days, while 35% had admissions exceeding 100 days. Most patients (80%) were already known to community Child and Adolescent Mental Health Services (CAMHS), and the Alliance Team was involved in gatekeeping decisions in 70% of cases. 60% of the young people did not have a recorded diagnosis of ADHD or ASD at the time of admission.

**Conclusion:**

The Alliance Team maintained a low Tier 4 admission rate (4.3%), with further improvement compared to previous years. Admissions were predominantly among older adolescents with high clinical complexity, which is reflected in an increased use of the Mental Health Act and prolonged inpatient stay. This shows the success of community-based crisis intervention in avoiding Tier 4 admissions, in all but the most severe of case. A substantial minority (35%) continued to be admitted out of area, likely due to ongoing system pressures. The high proportion of patients already known to CAMHS (80%) and involvement of the Alliance Team in gatekeeping decisions (70%) suggest opportunities for early collaborative intervention to reduce admissions. For future, Alliance Team should focus on improving support for 17-year-olds, improving clinician’s understanding of psychosis, and further evaluation of factors impacting length of stay and admission locality.

